# Seed-Specific Gene *MOTHER of FT* and *TFL1* (*MFT*) Involved in Embryogenesis, Hormones and Stress Responses in *Dimocarpus longan* Lour.

**DOI:** 10.3390/ijms19082403

**Published:** 2018-08-14

**Authors:** Yukun Chen, Xiaoping Xu, Xiaohui Chen, Yan Chen, Zihao Zhang, Xu Xuhan, Yuling Lin, Zhongxiong Lai

**Affiliations:** 1 Institute of Horticultural Biotechnology, Fujian Agriculture and Forestry University, Fuzhou 350002, China; cyk68@163.com (Y.C.); byxxp310107@163.com (X.X.); m18950589675_1@163.com (X.C.); spencer_cy@163.com (Y.C.); zhangzihao863@163.com (Z.Z.); xxuhan@163.com (X.X.); buliang84@163.com (Y.L.); 2 Institut de la Recherche Interdisciplinaire de Toulouse, IRIT-ARI, 31300 Toulouse, France

**Keywords:** *mother of FT and TFL1*, longan, embryogenesis, phytohormones, stress response, quantitative real-time PCR

## Abstract

*Mother of FT and TFL1* (*MFT*) belongs to phosphatidylethanolamine-binding protein (PEBP) family, which plays an important role in flowering time regulation, seed development, and germination. To gain insight into the molecular function of *DlMFT* in *Dimocarpus longan* Lour., we isolated *DlMFT* and its promoter sequence from longan embryogenic callus (EC). Bioinformatic analysis indicated that the promoter contained multiphytohormones and light responsive regulatory elements. Subcellular localization showed that the given the DlMFT signal localized in the nucleus, expression profiling implied that *DlMFT* showed significant upregulation during somatic embryogenesis (SE) and zygotic embryogenesis (ZE), and particular highly expressed in late or maturation stages. The accumulation of *DlMFT* was mainly detected in mature fruit and seed, while it was undetected in abortive seeds, and notably decreased during seed germination. *DlMFT* responded differentially to exogenous hormones in longan EC. Auxins, salicylic acid (SA) and methyl jasmonate (MeJa) suppressed its expression, however, abscisic acid (ABA), brassinosteroids (BR) showed the opposite function. Meanwhile, *DlMFT* differentially responded to various abiotic stresses. Our study revealed that *DlMFT* might be a key regulator of longan somatic and zygotic embryo development, and in seed germination, it is involved in complex plant hormones and abiotic stress signaling pathways.

## 1. Introduction

Longan (*Dimocarpus longan* Lour.) is a famous subtropical economic fruit tree in Southeast Asia. Embryogenesis is an important factor associated with fruit output quantity and quality in longan [1]. However, elucidating these mechanisms remains a challenge due to the limitations in accessibility of embryos in vivo [2]. In previous research, longan somatic embryogenesis (SE) was widely used as a model system to study the early development process of woody plants, especially their embryogenesis [1,3,4,5,6]. During plant SE, somatic cells undergo a series of morphological and biochemical changes and then differentiate into somatic embryos [7,8]. The development of SE closely resembles that of zygotic embryogenesis (ZE), it has been considered as a potential model for studying early events in plant embryo development [7], and considered as a model to study the tissular, cellular, and molecular properties of ZE [9,10].

The plant phosphatidylethanolamine binding protein (PEBP) family was divided into three main clades, *FLOWERING LOCUS T* (*FT*)-like, *TERMINAL FLOWER1* (*TFL1*)-like, and *MFT*-like [11,12,13,14]. *MFT*-like clade seems to be the phylogenetically ancestral to other clades [11,15,16]. *FT* and *TFL*1 are two regulators with antagonistic functions in controlling flowering time and plant architecture, *FT* acts as the floral activator, but *TFL1* acts as inhibitor [14,17,18,19]. Interestingly, swapping the single amino acid residues (His88/Asp144 in TFL1, Tyr85/Gln140 in FT) causes conversion of FT to TFL1 and vice versa [20]. Although the sequence of *MFT* shows high similarity to both *FT* and *TFL1*, the function of *MFT* in controlling flowering time might be different from *FT*/*TFL1*. Overexpression of *MFT* in *Arabidopsis* caused slightly early flowering under long days, but its function in determination of flowering time was redundant [21], whereas *PopMFT* did not identify a function in controlling flowering time [21,22]. Identification of a major QTL (quantitative trait loci) in *Arabidopsis* indicates *MFT* was a potential candidate gene for altered flowering time at an increased atmospheric (CO_2_) [23].

The expression pattern of *MFT* in several plants were very similar; *Arabidopsis thaliana MFT* [24], *Vitis vinifera MFT* [13], *Populus nigra MFT* [14], *Citrus unshiu MFT* [25], *Zea mays MFT* [26], *Triticum aestivum MFT* [27], *Jatropha curcas MFT* [28], *Glycine max MFT* [29] and *Hevea brasiliensis MFT1* [30] were mainly expressed in mature fruits or seeds. However, *Gossypium hirsutum MFT1* and *MFT2* were mainly expressed in flowers and low-transcribed in other tissues [31]. *SrMFT* were constitutively expressed in various tissues and developmental stages in *Symplocarpus renifolius*, although the expression of *SrMFT* in female-stage spadices was higher than in other stages, whereas overexpression *SrMFT* in *Arabidopsis* did not show the function in earlier flowering [32]. In addition, the expression of *AcMFT* showed a distinct decrease from the young frond stage to the sori development and maturation stages, and was regulated by photoperiod in *Adiantum capillus-veneris*, introduced *AcMFT* in *Arabidopsis* promoted flowering [33]. In *Arabidopsis*, *MFT* promoted embryonic growth during seed germination by directly repressing *ABI5* expression, it played a critical role in seed germination via a negative regulation of abscisic acid (ABA) signaling pathway [24], it also promoted fertility relevant to the brassinosteroid (BR) signaling pathway [34]. Meanwhile, *MFT* promoted primary dormancy during seed development, and enhanced germination in after-ripened imbibed seeds with exogenous ABA in *Arabidopsis* [35]. Conversely, *TaMFT*, an *MFT* homolog in wheat (*Triticum aestivum*) acted as a positive regulator of seed dormancy, while a negative regulator of seed germination [27]. The homolog of *MFT* in soybean responded to exogenous applied ABA and gibberellin (GA), ectopic expression of *GmMFT* in *Arabidopsis* did not affect the flowering time but suppressed seed germination [29]. Ectopic expression of *HbMFT1* in *Arabidopsis* inhibited seed germination, seedling growth, different with previous studies, it delayed flowering time [30]. *FvMFT* located in the nucleus and cytoplasmic membranes, ectopic expression of *FvMFT* in *Arabidopsis* also inhibited seed germination through integrating ABA and GA signaling, *FvMFT* could notably promote the primary root growth under sugar-deficient conditions [36].

At present, the expression profile of *MFT* during SE and ZE, and its signaling pathways involved in the response to hormones and environmental cues in embryogenic calli are still fragmented. In addition, the function of *MFT* homolog in longan has not been investigated. In our study, we isolated *DlMFT* and its promoter, and found that *DlMFT* was a seed-specific gene, and might play an important role in longan embryogenesis, seed development, and germination. *DlMFT* also involved in complex hormones and stress responses. The information provided by our study may help to illuminate the biological functions of *DlMFT* in longan.

## 2. Results

### 2.1. Isolation of DlMFT and Its Phylogenetic Analysis

**Figure 1:**
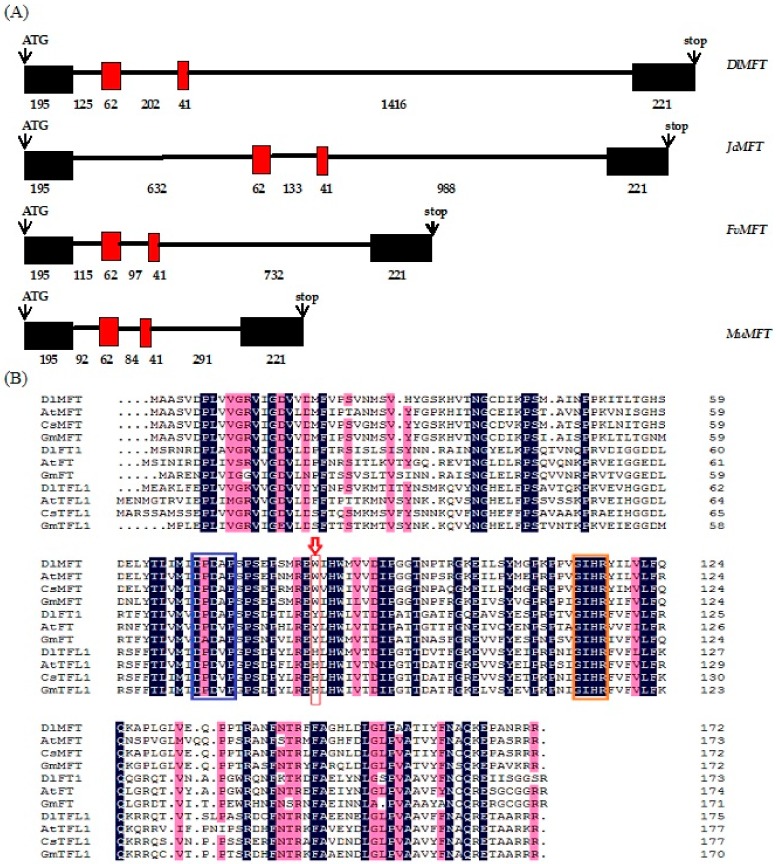
(**A**) Genomic organization analysis of *MFT* in plants. Coding regions (boxes) and introns (lines) are illustrated. Numbers represent the length of exons and introns (bp). (**B**) Multiple alignment of the PEBP proteins isolated from *Dimocarpus longan* (DlMFT, AKM77640.1; DlTFL1, AHZ89714.1; DlFT1, AHF27443.1), *Arabidopsis thaliana* (AtFT, AAF03936.1; AtMFT, AEE29676.1; AtTFL1,P93003.1), *Citrus sinensis* (CsMFT, XP_006490744.1; CsTFL1, BAH28255.1), *Glycine max* (GmFT, BAJ33494.1; GmMFT, AJF40168.1; GmTFL1, ACU00123.1), *Litchi chinensis* (LcFT, AEU08965.1). The red arrow indicates the critical amino acids distinguishing TFL1, FT, and MFT. Boxes indicate the highly conserved DPDxP and GIHR motifs. The background color represents the similarity of the PEBP proteins, the blue frame represents the DPDxP motif, the orange color represent the GIHR motif, the red arrow represent the key residues of PEBP proteins.

The RT-PCR (reverse transcription polymerase chain reaction) combined with RACE (rapid amplification of cDNA ends) strategy was used to obtain the complete cDNA (complementary DNA) and gDNA (genomic DNA) sequences of *DlMFT* from embryogenic callus of *Dimocarpus longan*. The amplified 866 bp region of *DlMFT* cDNA contained a 519 bp protein coding sequence flanked by a 54 bp 5′-untranslated region and a 312 bp 3′-untranslated region (GenBank accession No. KP861622). The gDNA sequence of *DlMFT* was cloned from longan EC DAN with specific primer and submitted to GenBank (GenBank accession No. KY968646), the comparative analysis of *MFT* genomic organization indicated that the *MFT* contained three introns and four exons, and owned the same number of nucleotides in exons (Figure 1a).

**Figure 2:**
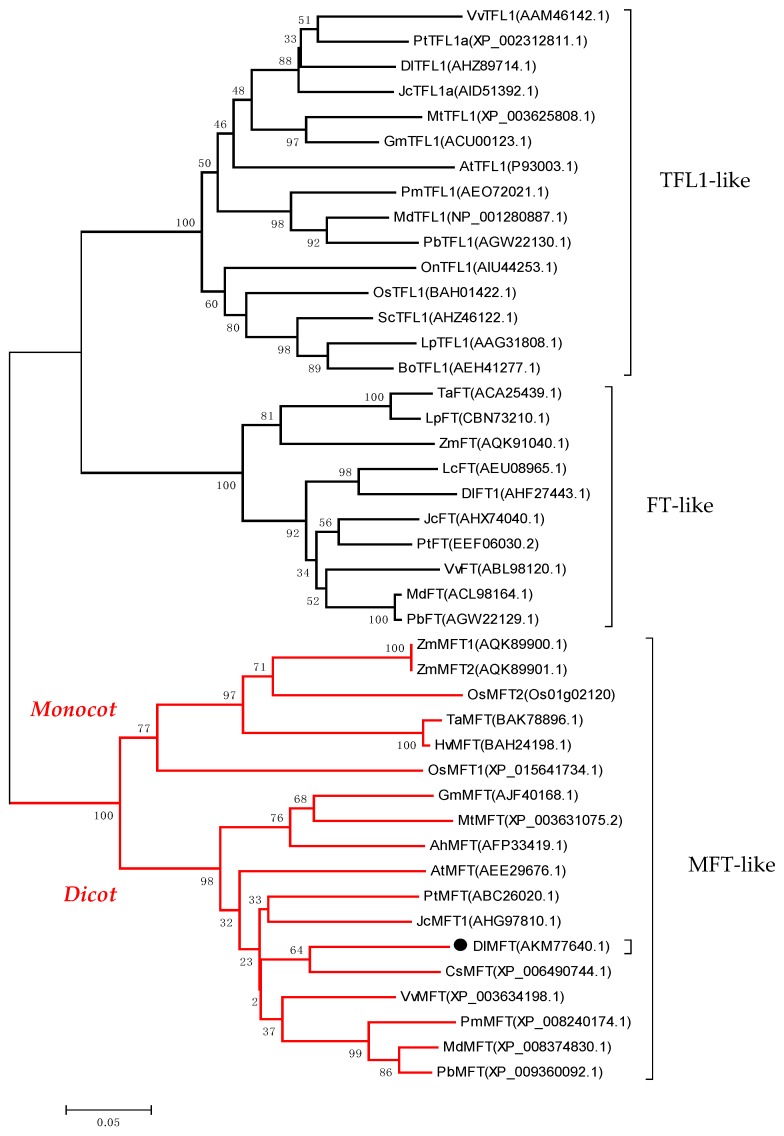
Phylogenetic tree of the PEPB proteins from plant. The number at the nodes represents the reliability percent of bootstraps values based on 1000 replications (%). The sequences are from *Dimocarpus longan* (DlMFT, AKM77640.1; DlTFL1, AHZ89714.1; DlFT1, AHF27443.1), *Vitis vinifera* (VvMFT, XP_003634198.1; VvTFL1, AAM46142.1; VvFT, ABL98120.1), *Zea mays* (ZmMFT1, AQK89900.1; ZmMFT2, AQK89901.1; ZmFT, AQK91040.1), *Oryza sativa* (OsMFT1, Os06g30370; OsMFT2, Os01g02120; OsTFL1, BAH01422.1), *Triticum aestivum* (TaMFT, BAK78908; TaFT, ACA25439.1), *Hordeum vulgare* (HvMFT, BAH24198), *Glycine max* (GmMFT, AJF40168.1; GmTFL1, ACU00123.1), *Medicago truncatula* (MtMFT, XP_003631075.2; MtTFL1, XP_003625808.1), *Arabidopsis thaliana* (AtMFT, AEE29676.1; AtTFL1,P93003.1), *Citrus sinensis* (CsMFT, XP_006490744.1), *Jatropha curcas* (JcMFT1, AHG97810.1; JcTFL1a, AID51392.1; JcFT, AHX74040.1), *Populus trichocarpa* (PtMFT, ABC26020.1; PtTFL1a, XP_002312811.1; PtFT, EEF06030.2), *Prunus mume* (PmMFT, XP_008240174.1; PmTFL1, AEO72021.1), *Malus domestica* (MdMFT, XP_008374830.1; MdTFL1, NP_001280887.1; MdFT, ACL98164.1), *Pyrus x bretschneideri* (PbMFT, XP_008374830.1; PbTFL1, AGW22130.1; PbFT, AGW22129.1), *Arachis hypogaea* (AhMFT, AFP33419.1), *Oncidium* (OnTFL1, AIU44253.1), *Saccharum* (ScTFL1, AHZ46122.1), *Lolium perenne* (LpTFL1, AAG31808.1; LpFT, CBN73210.1), *Bambusa oldhamii* (BoTFL1, AEH41277.1), *Litchi chinensis* (LcFT, AEU08965.1).

The DlMFT protein sequence shows 79%, 85%, and 86% identity with *Arabidopsis* MFT, *Malus domestica* MFT and *Citrus clementina* MFT, respectively (http://blast.ncbi.nlm.nih.gov). Two highly conserved DPDxP and GIHR motifs between the PEBP families were identified (Figure 1b). In *Arabidopsis*, the key residues Y (Tyr-85) and H (His-88) were possibly the most critical for distinguishing FT and TFL1 function on flowering regulation [12,13,37]. However, the key residue in DlMFT was W (Trp-83), leading to the hypothesis that *DlMFT* does not play a central role in flowering time regulation. A phylogenetic tree for the PEBP family was constructed using several amino acid sequences of other MFT orthologs (Figure 2). The PEBP family was divided into three clades: FT-like, TFL1-like and MFT-like, and DlMFT belonged to the eudicots group of the MFT clade and had the closest relationship with CsMFT from *Citrus sinensis*.

### 2.2. Isolation and Functional Analysis of the DlMFT Promoter

**Figure 3:**
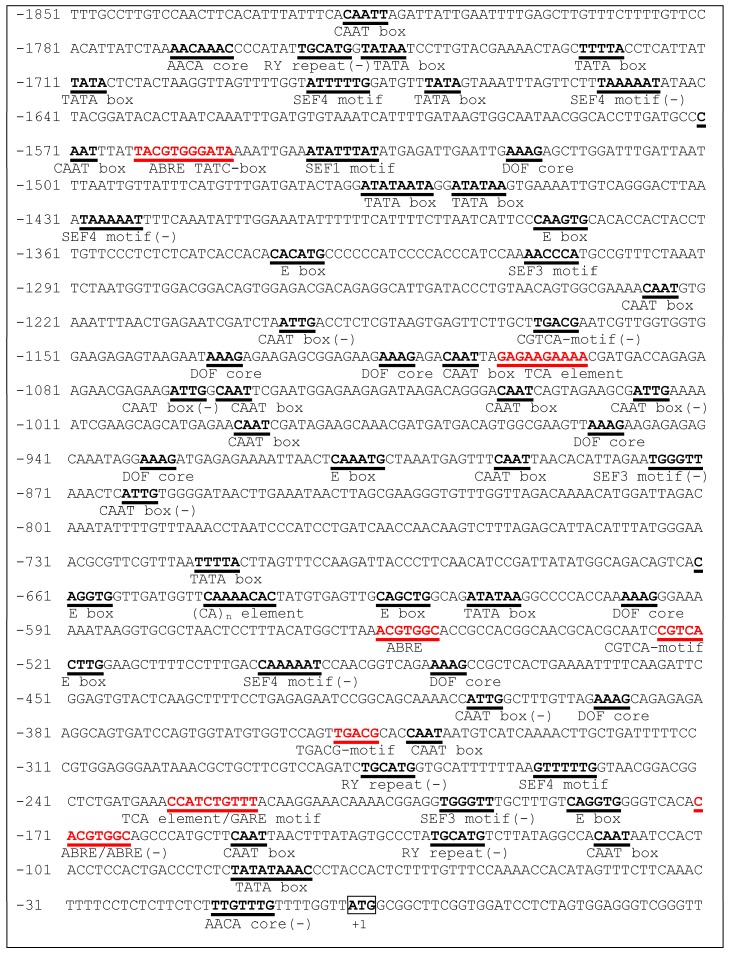
The nucleotide sequence of the *DlMFT* promoter. The A of the ATG (bold and boxed) is numbered as +1. The putative regulatory elements on both strands are shown in bold and underlined, the red colors indicate the hormone-responsive elements, which was performed by PlantCARE database.

Base on the gDNA sequence, a 1.85-kb *DlMFT* promoter fragment (GenBank accession No. KY860780) was isolated from embryogenic callus gDNA by genome walking. The PLACE database was used to analysis the putative cis-acting elements in this promoter, and the sequences on both strands were considered. The putative regulatory elements are show in Figure 3. The *DlMFT* promoter contained multiple regulatory elements, including the AACA core (AACAAAC), (CA)n element (CNAACAC), DOF core (AAAG), E box (CANNTG), RY repeat (CATGCA) and SEF1, SEF3, SEF4 motifs (1, ATATTTAWW; 3, AACCCA; 4, RTTTTTR), which were involved in seed-specific transcriptional regulation [28]. Meanwhile, the putative cis-acting elements was performed using the PlantCARE database, the promoter contains multiple regulatory elements in response to hormone signals, including five ABRE for ABA responsiveness, three CGTCA-motif/TGACG-motif for MeJa responsiveness, two TCA-element for SA responsiveness, TATC-box and GARE-motif for GA responsiveness. Therefore, the analysis results suggested that *DlMFT* may be regulated by ABA, GA3, SA and MeJa. Furthermore, there were 22 light-responsiveness elements, including GT1-motif (3), SP1 (3), GA-motif (3), GAG-motif (3), Box-I (2), G-box (2), ACE (1), AE-box (1), ATCT-motif (1), GATA-motif (1), I-box (1), and TCT-motif (1).

**Figure 4:**
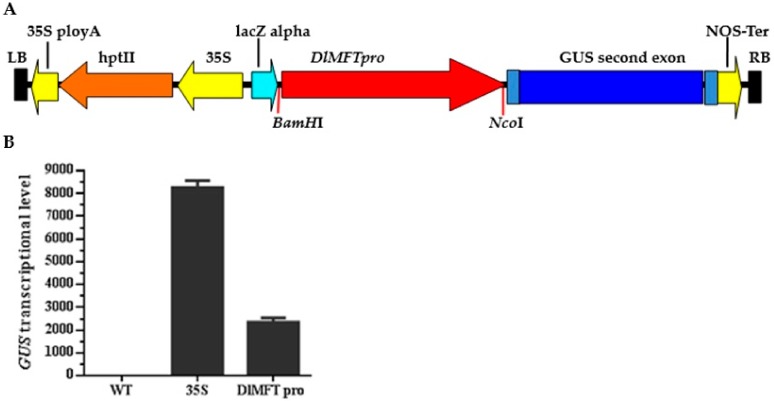
*GUS* transcriptional level generated by 35S promoter and *DlMFT* promoter in *N. benthamiana* (Reference gene *NbEF1a*). Data are means ± SD (*n* = 3). (**A**) Schematic diagram of the expression frame in *DlMFT pro:GUS* vector. (**B**) The relative expression of *GUS*.

The *DlMFT pro:GUS* fusion (Figure 4) was constructed for transformation in *N. Benthamiana*. Transient transformation of *DlMFT pro:GUS* in *Benthamiana* indicated that *DlMFT* promoter could generate the expression of *GUS*, and the activation of *DlMFT promoter* was 28.4% lower than 35S promoter. These suggested that the *DlMFT promoter* can be used for further studies.

### 2.3. Subcellular Localization of DlMFT

**Figure 5:**
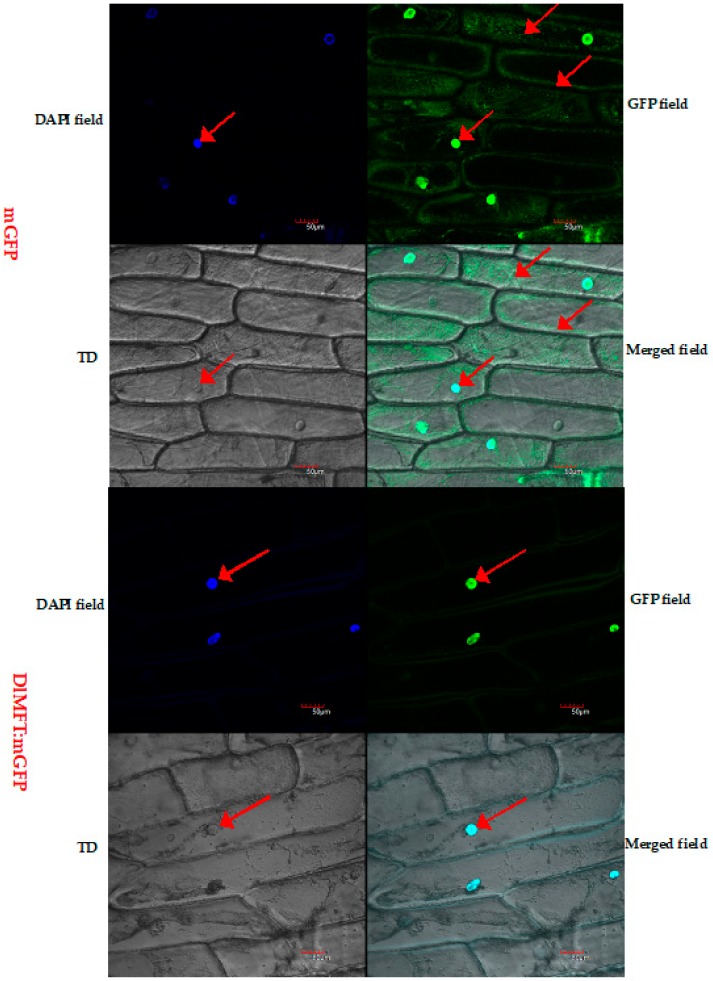
Subcellular localization of DlMFT. Transient expression of DlMFT:mGFP via agroinfiltration in onion (*Allium cepa*) scale leaf epidermis cells; the upper image is GFP fluorescence of mGFP (pCAMBIA1302), the lower image is GFP fluorescence of DlMFT:mGFP in scale leaf epidermis cells. TD, transmitted light channel, referring to the transmitted light differential interference contrast images of the cells. Bars = 50 μm. The arrow indicated that DlMFT was located in the nucleus. The green fluorescence was the GFP file, the deep blue fluorescence was the DAPI file, the light blue fluorescence was the Merged file.

The subcellular localization of DlMFT:mGFP was examined through a transient expression of DlMFT:mGFP in onion (*Allium cepa*) scale leaf epidermis cells, 4′,6-diamidino-2-phenylindol (DAPI) was used as a maker of nuclear localization. As shown in Figure 5, the green fluorescent signal of DlMFT:mGFP fusion protein was localized in the nucleus, while the GFP associated with positive control was dispersed throughout the cells. After DAPI staining, the blue fluorescent signal of DlMFT:mGFP and positive control all targeted to the nucleus. The result indicated that DlMFT was distributed in the nucleus.

### 2.4. DlMFT Played a Key Role during Longan SE and ZE, and Seed Germination

To better understand the transcription regulation of *DlMFT* during longan embryogenesis, its transcription profiles were analyzed by quantitative real-time PCR (qRT-PCR) during SE and ZE (Figure 7). As shown in Figure 7B, *DlMFT* was minimally expressed in non-embryogenic callus (NEC), embryogenic callus (EC) and incomplete compact pro-embryogenic cultures (ICpEC), but highly expressed in latter stages, and showed continuous up-regulation from ICpEC stage to cotyledonary embryos (CE) stages, and peaked at CE stage. Meanwhile, *DlMFT* mRNA was detected in all examined zygotic embryo development stages of ‘Honghezi’ longan (Figure 7A,C), its expression level was very low in S1 and S2 stages, and continuously increased during the embryo development stages, and showed a peak in mature zygotic embryo stage (S7). The results showed that *DlMFT* exhibited a similar expression pattern during longan SE and ZE, the expression profile implied a promotion role of *DlMFT* during longan SE and ZE, especially, at the late embryogenesis stages.

**Figure 6:**
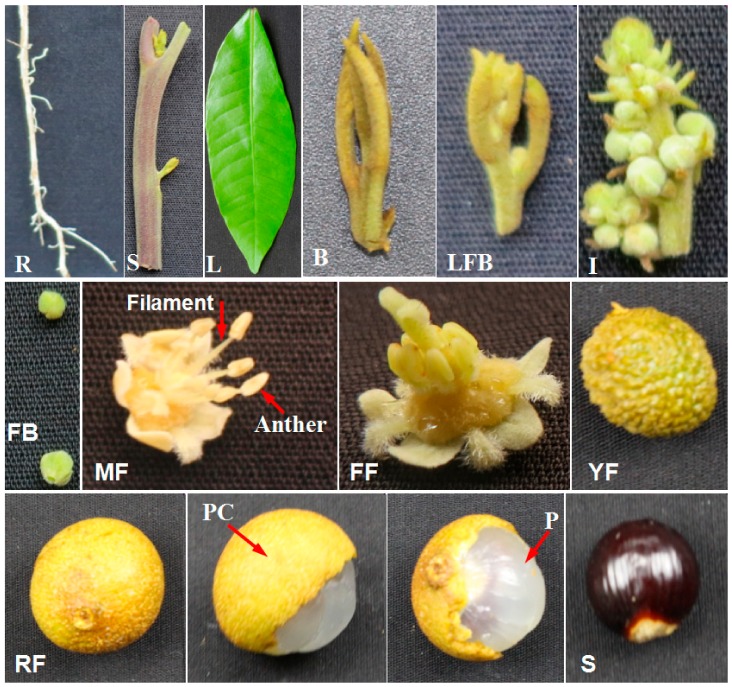
Different tissues of ‘Honghezi’ longan. R (root), S (stem), L (leaf), B (vegetative bud), LFB (late stage of floral bud), I (inflorescence), FB (flower bud), MF (male flower), FF (female flower), YF (young fruit), RF (ripe fruit), PC (pericarp), P (pulp), and S (seed).

**Figure 7:**
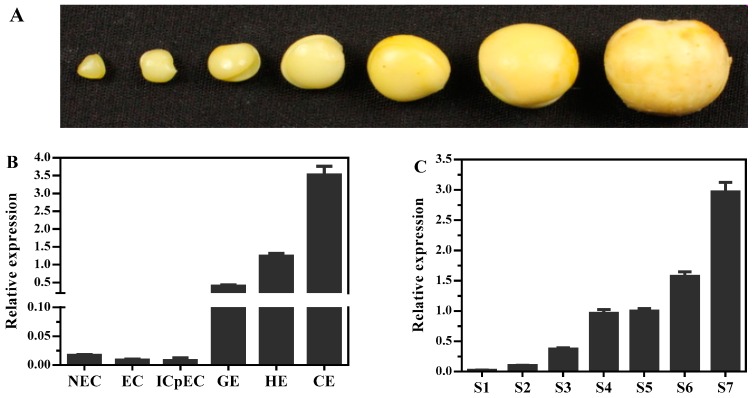

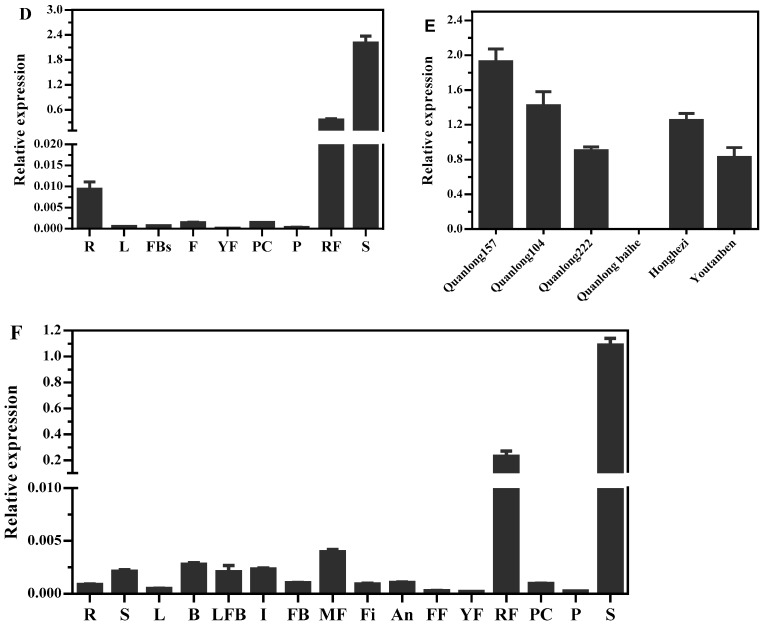
The expressions profile of *DlMFT* at different tissues and during longan embryogenesis. R: Root; S: Steam; L: Leaf; B: Vegetative bud; FB: Floral bud; I: Inflorescence; FBs: Flower buds; MF: Male flower; Fi: Filament; An: Anther; FF: Female flower; YF: Young fruit; RF: Ripe fruit; PC: Pericarp; P: Pulp; and S: Seed. (**A**). Different development stages of zygotic embryo, when the young fruits emerge cotyledon embryo stage was marked as S1, then collect the zygotic embryo every four days, marked as S2, S3, S4, S5, S6 and S7, ordinal. (**B**). Relative expression of *DlMFT* at different stages of longan somatic embryos. NEC: Non-embryogenic callus; EC: The friable-embryogenic callus; ICpEC: Incomplete compact pro-embryogenic cultures; GE: Globular embryos; HE: Heart embryos; CE: Cotyledonary embryos (Reference genes *EF1a*, *FSD*, *elf4a*); (**C**). Relative expression of *DlMFT* at different stages of longan embryo (Reference genes *EF1a*, *2-TU*, *UBQ*); (**D**). Relative expressions of *DlMFT* at different tissues of ‘Sijimi’ longan (Reference genes *EF1a*, *FSD*, *elf4a*). (**E**). Relative expressions of *DlMFT* at different species of longan seeds (Reference genes *FSD*, *ACTB*, *18S*). (**F**). Relative expressions of *DlMFT* at different tissues of ‘Honghezi’ longan (Reference genes *EF1a*, *ACTB*, *18S*). Data are means ± SD (*n* = 3).

To gain insight into the expression profile of *DlMFT* in longan, we examined the levels of *DlMFT* transcript in different tissues, including roots, stems, leaves, vegetative buds, floral buds, inflorescence, flower buds, male flowers, filaments, anthers, female flowers, young fruits, ripe fruits, pericarp, pulp and seeds of ‘Honghezi’ longan (Figure 6); and the roots, leaves, flower buds, flowers, young fruits, ripe fruits, pericarp, pulp and seeds of ‘Sijimi’ longan. In ‘Honghezi’ longan (Figure 7F), *DlMFT* was highly expressed in the ripe fruits and seeds, however, it was minimal or undetected in other tissues, including the pericarp and pulp. Similarly, *DlMFT* also showed the ripe fruits and seeds specific in ‘Sijimi’ longan (Figure 7D), which revealed that *DlMFT* was a seed-specific gene. Furthermore, we harvested the normal and abortive seeds from their ripe fruits of different species at the same days (100 day) after flowering, and checked the expression of *DlMFT* in these seeds. The result showed that *DlMFT* was highly expressed in normal development seeds, but was undetected in abortive seeds of ‘Quanlong baihe’ longan (Figure 7E). Hence, we concluded that *DlMFT* played an important role in controlling the embryo and seed development.

**Figure 8:**
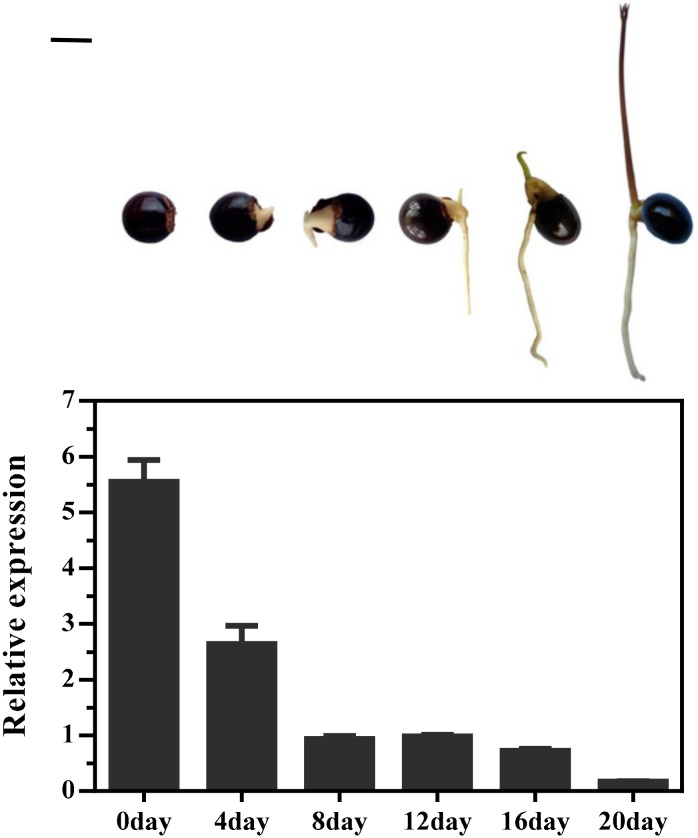
Relative expressions of *DlMFT* during longan seeds germination (Reference genes *EF1a*, *FSD*, *18S*). Data are means ± SD (*n* = 3). Bar = 1 cm.

In addition, the expression pattern of *DlMFT* was analyzed during seed germination processes (0, 4, 8, 12, 16 and 20 day) with quantitative real-time PCR. The expression level of *DlMFT* was significantly decreased at 4-day and 8-day periods, and then kept at a relatively stable and low level at 8, 12 and 16 day periods, finally markedly decreased at 20-day period (Figure 8), which suggested that *DlMFT* appeared to be a negative regulator of seed germination.

### 2.5. The Effect of Exogenous Hormones on DlMFT Expression

**Figure 9:**
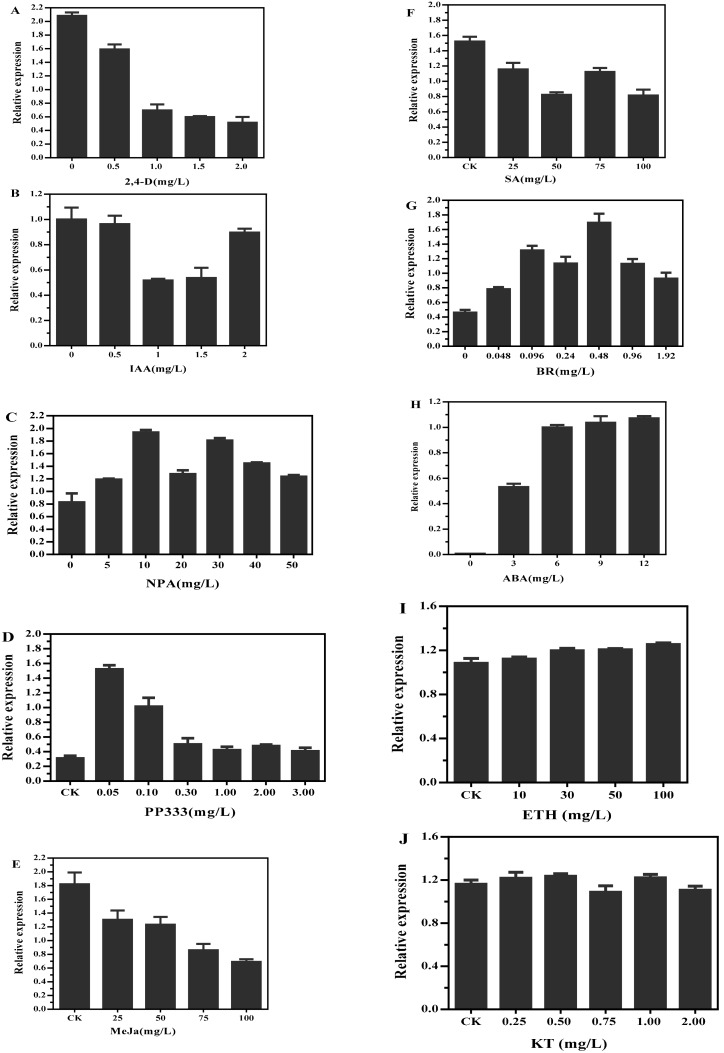
Relative expression of *DlMFT* transcripts in response to exogenous hormones. EC was treated with nutrient solution in the presence of the listed hormones at the indicated concentrations (MS 0 as control). The *DlMFT* expression level was normalized to *EF-1a*, *elf4a*, and *FSD*. (**A**) Treatment with 2,4-D (0.5, 1.0, 1.5, and 2.0 mg/L); (**B**) Treatment with IAA (0.5, 1.0, 1.5, and 2.0 mg/L); (**C**) Treatment with NPA (5, 10, 20, 30, 40 and 50 mg/L); (**D**) Treatment with PP333 (0.05, 0.1, 0.3, 1.0, 2.0 and 3.0 mg/L); (**E**) Treatment with MeJa (25, 50, 75, and 100 mg/L) (**F**) Treatment with SA (25, 50, 75, and 100 mg/L); (**G**) Treatment with BR (0.048, 0.096, 0.24, 0.48, 0.96 and 1.92 mg/L); (**H**) ABA at the indicated concentrations (3, 6, 9 and 12 mg/L). Data are means ± SD (*n* = 3).

In an attempt to understand whether *DlMFT* is a hormone responsive gene, we analyzed the transcription of *DlMFT* in longan EC under different concentration of 2,4-D, indole-3-acetic acid (IAA), auxin transport inhibitor (NPA), PP333, MeJa, SA, BR, ABA, kinetin (KT) and ethylene (ETH) for 24 h. Among the treatments, exogenous 2,4-D inhibited its expression, and higher concentration of 2,4-D induced a more significantly expression decline (Figure 9A); IAA at the concentrations of 1.0 and 1.5 mg/L reduced the *DlMFT* transcript level, by about 1.9-fold compared with the control, but only a slight effect at 0.5 and 2.0 mg/L IAA was observed (Figure 9B). The expression of *DlMFT* was continuously declined with higher concentration of MeJa (Figure 9E), meanwhile, *DlMFT* mRNA was also suppressed by SA, under 50 and 100 mg/L SA treatments, its expression level drastically reduced by about 54% compared with the control (Figure 9F). Interestingly, ABA, NPA and BR treatments generated an opposite effect on *DlMFT* transcript level compared with the 2,4-D, IAA, MeJa and SA treatments; 10 mg/L NPA and 0.48 mg/L BR most obviously enhanced its transcript level (Figure 9C,G). 0.05 and 0.1 mg/L growth retardator (PP333) increased the expression approximately 4.9-fold and 3.3-fold, but when concentration higher than 0.1 mg/L it offset the positive effects (Figure 9D). As shown in Figure 9H, 3 and 6 mg/L ABA enhanced the *DlMFT* transcript significantly, when the concentration was higher than 6 mg/L, this promotion was only slightly improved. In addition, KT and ETH had no significantly effect on *DlMFT* transcript level (Figure 9I,J).

Taken together, our results revealed the transcription mechanism of *DlMFT* in responding to different exogenous hormones in longan EC. The expression of *DlMFT* was negatively regulated by auxins, MeJa and SA, while auxin inhibitor (NPA), ABA, BR and PP333 did the opposite function, and KT and ETH showed no significantly function on *DlMFT* expression.

### 2.6. The Effects of Light and Abiotic Stress on DlMFT Expression

**Figure 10:**
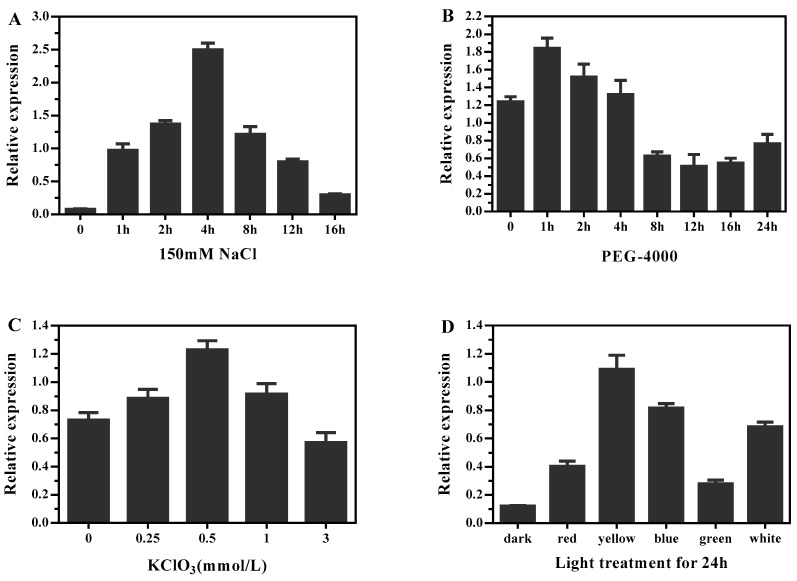

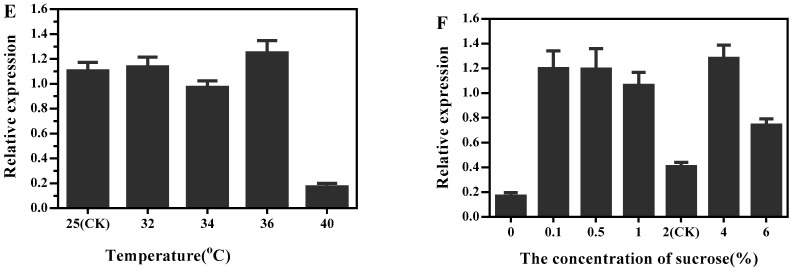
*DlMFT* expression in response to abiotic stress. EC was treated with nutrient solution containing 150 mM NaCl or PEG-4000 for different times, KClO_3_ at the indicated concentrations, or exposed to different light qualities. RNA was isolated from samples treated with: (**A**) 150 mM NaCl (0, 1, 2, 4, 8, 12 and 16 h), (**B**) PEG-4000 (0, 1, 2, 4, 8, 12, 16 and 24 h), (**C**) different light qualities (dark, red, green, blue, and white) for 24 h, (**D**) KClO_3_ (0.25, 0.5, 1 and 3 mM/L), (**E**) different temperature (25, 32, 34, 36 and 40 °C), (**F**) sucrose (0, 0.1, 0.5, 1.0, 2.0, 4.0 and 6.0%). Reference genes were *EF1a*, *FSD*, and *elf4a*. Data are means ± SD (*n* = 3).

In order to determine whether the transcriptional regulation of *DlMFT* is affected by abiotic stress, we detected the mRNA level of *DlMFT* in longan EC exposed to 150 mM NaCl and PEG-4000 for different time, and treated with different concentration of sucrose, KClO_3_ at 110 rpm at 25 °C under dark conditions for 24 h. In addition, longan EC was treated under different temperature and light qualities conditions for 24 h to define whether *DlMFT* responded to temperature and light. The results revealed that treatment with 150 mM NaCl for 1, 2, and 4 h induced a significant expression increase as the treatment time increased, and the highest transcript level (~32.4-fold of the control) of *DlMFT* was observed at 4 h; then, as the treatment time increased, the transcript level declined, but still higher than the control (Figure 10A). In the PEG-4000 treatments, no more than 4 h treatment time slightly enhanced the transcript level of *DlMFT*. In contrast, when treatment time was longer than 4 h the transcript level obviously declined (Figure 10B). As Figure 10C showed, KClO_3_ treatment promoted the expression of *DlMFT*, except 3 mM treatment, and the maximum expression of *DlMFT* mRNA at 0.5 mM treatment. When treated with different light qualities, the highest transcript level of *DlMFT* was observed in yellow light, approximate 9.1-fold of the control, the following was blue, white, red and green (Figure 10D). The expression of *DlMFT* at 32, 34 and 36 °C treatments were similarly to that at 25 °C (CK). However, it showed an arresting decline in the 40 °C treatment (Figure 10E). Interestingly, compared to the control (2% sucrose), higher or lower concentration of sucrose was beneficial to increase the transcript level of *DlMFT*, but sugar-free treatment showed an opposite effect (Figure 10F). The results indicated that *DlMFT* mRNA differently responded to salinity stress, PEG-4000, KClO_3_, temperature and sucrose stress. Besides, *DlMFT* was a light-responsive gene—the accumulation of *DlMFT* mRNA was stimulated by various light qualities.

## 3. Discussion

### 3.1. DlMFT Played a Key Role in Longan Embryogenesis and Seed Germination

Although *MFT* shows high similarity in sequence to *FT* and *TFL1*, the function of *MFT* was different with *FT*/*TFL1*. To date, the functional studies of *MFT* were mainly focus on expression pattern, flowering time control and seed germination. Previous studies had proved that *MFT* from different plants showed different functions on flowering time, for example, ectopic expression of *AtMFT* [21] and *AcMFT* [33] in *Arabidopsis* promoted flowering. However, *PopMFT* [22], *SrMFT* [32], and *GmMFT* [29] had no effect on flowering, and *HbMFT1* [30] even delayed flowering when it was introduced in *Arabidopsis*. The recent researches on *MFT* revealed that most of the *MFT* showed preferential expression in seeds, and mainly involved in seed development and germination. In *Arabidopsis*, *AtMFT* promoted seed germination [24,35], fertility relevant to BR signaling pathway [34], and primary seed dormancy and germination [35]. By contrast, *TaMFT* [27], *FvMFT* [36], *HbMFT1* [30], *GmMFT* [29] suppressed seed germination, opposite to the action of *AtMFT*. In our study, *DlMFT* showed distinct seed-specific expression pattern, and showed similar upregulated expression during longan SE and ZE processes, especially, highly expressed at the late or mature embryo stages. However, *DlMFT* was not detected in abortive longan seeds, but highly expressed in normal development seeds. During seed germination, *DlMFT* was significantly downregulated. Hence, we speculated that *DlMFT* could served as a key mediator to regulate longan SE and ZE, as well as seed germination.

### 3.2. DlMFT Was Involved in Various Plant Hormone Signaling Pathways during Longan SE

As we discussed above, *MFT* play an important role in plant embryo development and seed germination through several hormone-signaling pathways. For instance, Xi et al. found that *AtMFT* regulated seed germination through a negative feedback loop modulating ABA signaling in *Arabidopsis*, *AtMFT* was regulated by DELLA protein [24]. During seed germination, *GmMFT* was inhibited by ABA, but promoted by GA3, ectopic expression of *GmMFT* in *Arabidopsis* affected the expression of ABA and GA metabolism and signaling genes [29]. Ectopic expression of *FvMFT* in *Arabidopsis* also inhibited seed germination through integrating ABA and GA signaling [36]. Furthermore, *AtMFT* promoted fertility through the BR signaling pathway [34], *DlMFT* also promoted by BR. Hence, *MFT* played an important role in seed development and germination via ABA, GA, and BR signaling pathway. However, very few studies focus on the plant hormones in regulating *MFT* during SE.

In our study, *DlMFT* was inhibited by exogenous 2,4-D, IAA, while promoted by auxin transport inhibitor (NPA) and plant growth inhibitor (PP333) in longan EC. During longan SE, high dose of 2,4-D was essential for SE induction [4], 1.0 mg/L 2,4-D was the key factor of EC long term maintance with growing vigorously [3]. The level of endogenous IAA in early SE was higher than that in NEC, while decrease after GE stage till CE stage [2]. In our study, *DlMFT* accumulation facilitated the morphogenesis in the middle and late SE and ZE stages, which suggested that *DlMFT* might involved in auxin signaling pathway during longan SE. ABA is an important plant growth regulator that is involved in embryo formation and maturation [38,39,40]. During SE, ABA not only contributed to normal morphological development of embryos [41,42], but also involved in the improvement of somatic embryo quality for many species by promoting desiccation tolerance and inhibiting precocious germination [43,44,45,46]. In our study, *DlMFT* was promoted by ABA, consistent with the study that the expression of *AtMFT* was induced by ABA during seed germination [24]. Plus the fact that endogenous ABA was increased during longan SE, especially at the late embryonic stages [2]. Meanwhile, *DlMFT* was promoted by exogenous GA3, and inhibited by SA and MeJa. In addition, *DlMFT* promoter contained multiple elements related to hormone signals, including ABRE for ABA responsiveness, CGTCA-motif for MeJa responsiveness, TCA-element for SA responsiveness, TATC-box and GARE-motif for GA responsiveness. Thus, it was suggested that *DlMFT* was involved in various hormones signaling during longan SE.

### 3.3. DlMFT Participated in Responses to Various Abiotic Stresses

Stress acts as an embryogenic stimulus, which plays an important role in plant SE [47]. In citrus, desiccation and low-temperature treatments were beneficial to promote SE, resumed and enhanced the capacity of embryogenesis [48]. It was reported that 25 mM sodium chloride could enhance callus proliferation, somatic embryo formation and conversion in date palm, but a negative effect was found by higher dose of NaCl [49]. Our results also showed that certain treatment time of PEG-4000, NaCl and KClO_3_ could increase the accumulation of *DlMFT*. Relatively high temperature was effective to induce secondary somatic embryos, while a low temperature was more suitable for further embryo development [50]. The expression of *DlMFT* was inhibited with 40 °C treatment, while no significant change of the expression was found when treated with 32, 34 and 36 °C.

Light and temperature are the key abiotic modulators of plant gene expression [51]. Light qualities were employed to significantly enhance SE in many species [39,52,53,54,55,56]. In this study, observed that there were 22 light responsive elements (GT1-motif, SP1, GA-motif, GAG-motif, Box-I, G-box, ACE, AE-box, ATCT-motif, GATA-motif, I-box, and TCT-motif) in the 5′ flanking sequence of *DlMFT* were observed, which might involve in transcriptional regulations of *DlMFT* gene. We found that *DlMFT* responded differently to various light treatments, all the light treatments up-regulated the expression of *DlMFT*, especially, with the yellow and blue light treatments. These results indicated that *DlMFT* might be involved in complicated light signaling pathways during longan SE.

In addition, sugars have important hormone-like functions as primary messengers in signal transduction, sugar signaling is yet another potential source for regulation of nuclear genes [57]. High concentration of sucrose generated osmotic stress in culture medium, and played an important role in promoting the maturation of somatic embryos during longan SE [2]. Over-expression of *FvMFT* in *Arabidopsis* notably promoted the primary root growth under sugar-deficient condition [36], which suggested that *MFT* might be involved in sugar signaling. In the present research, compared with 2% sucrose (control), the transcription level of *DlMFT* was promoted at higher and lower concentration of sucrose, while inhibited under sucrose-deficient condition, which indicated that *DlMFT* was included in sugar signaling pathway. All these results indicate that *DlMFT*, at least in part, take part in the abiotic stress response.

## 4. Materials and Methods

### 4.1. Plant Materials and Treatments

The synchronized embryogenic cultures at different developmental stages, including the non-embryogenic callus (NEC), friable-embryogenic callus (EC), incomplete compact pro-embryogenic cultures (ICpEC), globular embryos (GE), heart-shape embryos (HE), cotyledonary embryos (CE) were obtained by the following previously published methods [2,4]. ‘Honghezi’ Longan tissues or organs for qRT-PCR were showed at Figure 6. Different development stages of zygotic embryo were collected for qRT-PCR, when the young fruits emerge cotyledon embryo stage was marked as S1, then collect the zygotic embryo every four days, marked as S2, S3, S4, S5, S6 and S7, ordinal (Figure 7a).

Transferred 0.2 g 18-day subculture longan EC to MS liquid basal medium (2% sucrose) supplemented with ABA (3, 6, 9, and 12 mg/L) (Solarbio, Beijing, China), 2,4-D (0.5, 1.0, 1.5, and 2.0 mg/L) (TCI, Shanghai, China), BR (0.1, 0.2, 0.5, 1.0, 2.0, and 4.0 μM) (Yuanye, Shanghai, China), IAA (0.5, 1.0, 1.5, and 3.0 mg/L) (Sigma-Aldrich, SAFC, USA), SA (25, 50, 75, 100mg/L) (SCR, Shanghai, China), MeJa (25, 50, 75, 100 mg/L) (Aladdin, Shanghai, China), GA3 (3, 6, 9, and 12 mg/L) (YEASEN, Shanghai, China), *N*-1-Naphthylphthalamic acid (NPA: 25, 50, 75, and 100 μM) (Solarbio, Beijing, China), Paclobutrazol (PP333: 25, 50, 75, and 100 μM) (Solarbio, Beijing, China), KClO_3_ (0.25, 0.5, 1.0, and 3.0 mM) (SCR, Shanghai, China) with agitation at 110 rpm at 25 °C under dark conditions for 24 h, with 3 replicates. EC culture in MS liquid medium as control. EC treated with 1 mg/L IAA for different time (4, 8, 12, 16, 20 and 24 h), treated with 150 mM NaCl for different time (1, 2, 4, 8, 12 and 16 h) and treated with PEG-4000 for different time (1, 2, 4, 8, 12, 16 and 24 h) in liquid MS medium with agitation at 110 rpm at 25 °C under dark conditions, EC culture in MS liquid medium as control. EC treated with different light qualities (dark, red, yellow, blue, green and white) was carried out at 25 °C for 24 h. Frozen all samples in liquid nitrogen immediately for 5 min after collecting, then stored at −80 °C for isolating the total RNA.

### 4.2. Isolation of DlMFT and Its Promoter

The cDNA was synthesized from total RNA of embryo callus using GeneRacer™ Kit (RLM-RACE) (Invitrogen, Carlsbad, CA, USA) according to the manufacturer’s protocol. Base on the sequence of *MFT* from longan embryo callus transcriptome (SRR accession number: SRA050205), specific primers *DlMFT-GSP1* and *DlMFT-GSP2* couple with 3′-primer and 3′-Nest primer, respectively, were used for 3′-RACE, *DlMFT-GSPa* and *DlMFT-GSPb* couple with 5′-primer and 5′-Nest primer, respectively, were used for 5′-RACE, *DlMFT*-F/R were used for Splice verification and gDNA amplification, all primers are list in Table 1. All amplifications were performed with the following parameters: 94 °C for 3 min (94 °C for 30 s, 54 °C for 30 s and 72 °C for 1 min) for 35 cycles, 72 °C for 10 min, but different primer with some modifications. Purified the PCR products by agarose gel electrophoresis, then cloned into pMD 18-T vector (Takara, Mountain View, CA, USA) and sequenced.

Based on the gDNA sequence of *DlMFT*, specific primers SP1, SP2 and SP3 and the adaptor primers AP1 and AP2 were used for cloning the promoter by Tail-RCR (thermal asymmetric interlaced PCR) protocol of genome walking Kit (Takara) from embryogenic callus DNA. The PLACE (https://sogo.dna.affrc.go.jp/cgi-bin/sogo.cgi?lang=en&pj=640&action=page&page=newplace) database and the PlantCARE (http://bioinformatics.psb.ugent.be/webtools/plantcare/html/) database were used to analyze the putative cis-acting regulatory elements. The primers used in promoter cloning are list in Table 1.

### 4.3. Subcellular Localization of DlMFT Protein

In order to generate DlMFT: GFP fusion protein, the coding sequence (CDS) of *DlMFT* without a terminator codon (TAA) was sub-cloned in *Bgl*II sites of pCAMBIA1302-GFP vector for investigating the subcellular localization of DlMFT in *Allium cepa*. The pCAMBIA1302-GFP vector (served as the positive control) was co-transformed into *Allium cepa*. 4′,6-diamidino-2-phenylindole (DAPI) was used as a maker of nuclear localization. Transient transformation of onion (*Allium cepa*) scale leaf epidermis cells was performed using the agrobacterium-mediated gene expression system and analyzed by laser scanning con-focal microscopy (Olympus; FV1200, Tokyo, Japan). The protocols were similar to transient transformation of onion scale leaf epidermal cells described previously [58], with some modifications.

### 4.4. Quantitative Real-Time PCR Analysis

Total RNA of longan embryogenesis cultures and EC under various treatments were extracted by means of TriPure Isolation Reagent (Roche Diagnostics, Indianapolis, IN, USA). Total RNA was isolated from longan tissues and different germination stages with the Universal Plant Total RNA Extraction Maxi Kit (Spin-column) (Bioteke Corp., Beijing, China). Total RNA of *N. Benthamiana* were extracted with TransZol Up Plus RNA Kit (TransGen, Beijing, China). Samples were then treated with DNase I to remove any genomic DNA. RNA was quantified by NanoDrop Lite spectrophotometer (Thermo Electron Corp., Houston, TX, USA) and checked the integrality using 1.0% agarose gel electrophoresis.

The cDNA was synthesized with a PrimeScript RT reagent Kit (Takara), the amount of RNA was 500 ng in 10 μL reaction system. qRT-PCR was performed on the Lightcycler 480 system (Roche Applied Science, Basel, Switzerland) in a 20 μL final volume containing 10 μL of 2× SYBR Premix Ex Taq^TM^ (Takara), 1 μL of 10× diluted cDNA, and 0.8 μL specific primer pairs (100 nM, listed in Table 1), and 7.4 μL of ddH_2_O. *EF-1α*, *eIF-4α*, and *Fe-SOD* were used as internal controls for calculating the relative expression of *DlMFT* at different stages of SE [59], different treatments and different tissues of ‘Sijimi’ longan as described previously [5,59]. The expression of *DlMFT* during longan seeds germination stages was normalized to the expression of longan *EF1a*, *FSD*, *18S* reference genes. *EF1a*, *ACTB*, *18S* were used as reference genes for calculating the relative expression of *DlMFT* at different tissues of ‘Honghezi’ longan. Expression levels of specific genes in *N. benthamiana* were normalized to *NbEF1a*. The primers used in this study were listed in Table 1.

### 4.5. DlMFT Pro:GUS Plasmid Construction and N. Benthamiana Transformation

To generate the *DlMFT pro:GUS* plasmid, a 1.85-kb *BamH*I (5′)-*Nco*I (3′) *DlMFT* promoter fragment was subcloned into the *BamH*I-*Nco*I site of pCAMBIA1301, and designated this recombinant plasmid as *DlMFT pro:GUS,* used pCAMBIA1301 as control, then transferred *DlMFT pro:GUS* and pCAMBIA1301 into *A. tumefaciens EHA105*, respectively. Transient transformed the resulting *A. Tumefaciens EHA105* in *N. Benthamiana* leaves by the injection method, then cultured the transformation plant in the greenhouse at 25 °C under dark conditions for three days. The injected part of the leaves was then harvested for verifying the activity of 35S and *DlMFT* promoter by qRT-PCR.

## Figures and Tables

**Table 1:** Primers Used in Cloning, qRT-PCR, and Vector Constructions.

Primer Name	Primer Sequence (5′–3′)	Description
*DlMFT 3′GSP1*	GGATTCACTGGATGGTCGT	3′-RACE
*DlMFT 3′GSP2:*	TCAACAGAAGGCACCATTAG	
*DlMFT 5′GSPa*	CCTAATGGTGCCTTCTGTTG	5′-RACE
*DlMFT 5′GSPb*	GACCATCCAGTGAATCCATT	
*DlMFT-F*	TTTTGGTTATGGCGGCTTC	Splice verification, gDNA amplification
*DlMFT-R*	ATGCTCCAGACCCCATACTA	
*DlMFT-SP1*	GCGAACCAGTGAATCCATT	Promoter cloning
*DlMFT-SP2*	CATCAGAGTGACCAGTGAGAGT	
*DlMFT-SP3*	TGATGTCACAGCCGTTGGT	
*DlMFT-pro-F*	GCGGATCCTTTGCCTTGTCCAACTTCAC	For construction of *DlMFT pro:GUS*. The added *Bam*I (GGATCC) and *Nco*I (CCATGG) sites were underlined
*DlMFT-pro-R*	GCCCATGGCAAACAAAGAGAAGAGAGGA	
*DlMFT-SL-F*	GAAGATCTATGGCGGCTTCGGTGGATC	Subcellar localization of DlMFT. The added *Bgl*II (AGATCT) site was underlined
*DlMFT-SL-R*	GAAGATCTGCGGCGACGGTTGGCCTTCC	
*DlMFT-q-F*	AACGGCTGTGACATCAAGC	qRT-PCR
*DlMFT-q-R*	CGACCATCCAGTGAATCCA	
*NbEF1a-F*	AGAGGCCCTCAGACAAAC	Reference gene for qRT-PCR in *N. benthamiana*
*NbEF1a-R*	TAGGTCCAAAGGTCACAA	
*GeneRacer^TM^ 3′-Primer*	GCTGTCAACGATACGCTACGTAACG	3′-RACE
*GeneRacer^TM^ 3′-Nested Primer*	CGCTACGTAACGGCATGACAGTG	
*GeneRacer^TM^ 5′-Primer*	CGACTGGAGCACGAGGACACTGA	5′-RACE
*GeneRacer^TM^ 5′-Nested Primer*	GGACACTGACATGGACTGAAGGAGTA	

## Abbreviations

*Dl*	*Dimocarpus longan*
*MFT*	*mother of FT and TFL1*
*FT*	*Flowering locus T*
*TFL1*	*terminal flowering like 1*
SE	somatic embryogenesis
ZE	zygotic embryogenesis
qRT-PCR	quantitative real-time PCR
NEC	non-embryogenic callus
EC	embryogenic callus
ICpEC	incomplete compact pro-embryogenic cultures
GE	globular embryos
TE	torpedo-shaped embryos
CE	cotyledonary embryos
2,4-D	2,4-Dichlorophenoxyacetic acid
IAA	indoleacetic acid
ABA	abscisic acid
GA	gibberellin
SA	salicylic acid
MeJa	methyl jasmonate
BR	brassinosteroids
NPA	*N*-1-Naphthylphthalamic acid
PP333	Paclobutrazol
GFP	green fluorescent protein
35S	CaMV35S
DAPI	4′,6-diamidino-2-phenylindol
